# Broad Auto-Reactive IgM Responses Are Common In Critically Ill COVID-19 Patients.

**DOI:** 10.21203/rs.3.rs-128348/v1

**Published:** 2020-12-31

**Authors:** Cheryl Maier, Andrew Wong, Isaac Woodhouse, Frank Schneider, Deanna Kulpa, Guido Silvestri

**Affiliations:** Emory University; Emory University; Oxford University; Emory University; Department of Pediatrics, Emory University School of Medicine, Atlanta, GA; Division of Microbiology and Immunology, Yerkes National Primate Research Center, and Emory Vaccine Center Emory University, Atlanta, GA

**Keywords:** COVID-19, immunoglobulin M (IgM), pathogenesis

## Abstract

The pathogenesis of severe COVID-19 remains poorly understood. While several studies suggest that immune dysregulation plays a central role, the key mediators of this process are yet to be defined. Here, we demonstrate that plasma from a high proportion (77%) of critically ill COVID-19 patients, but not healthy controls, contains broadly auto-reactive immunoglobulin M (IgM), and only infrequently auto-reactive IgG or IgA. Importantly, these auto-IgM preferentially recognize primary human lung cells in vitro, including pulmonary endothelial and epithelial cells. By using a combination of flow cytometry, LDH-release assays, and analytical proteome microarray technology, we identified high-affinity, complement-fixing, auto-reactive IgM directed against 263 candidate auto-antigens, including numerous molecules preferentially expressed on cellular membranes in pulmonary, vascular, gastrointestinal, and renal tissues. These findings suggest that broad IgM-mediated autoimmune reactivity may be involved in the pathogenesis of severe COVID-19, thereby identifying a potential target for novel therapeutic interventions.

Although SARS-CoV-2, the etiological agent for COVID-19, is initially and preferentially tropic for respiratory cellular targets^3–5^, its pathogenetic effects can be systemic. Indeed, dysregulated coagulopathy and systemic inflammation are hallmark characteristics of severe COVID-19^6,7^, which involves acute respiratory distress syndrome (ARDS) as well as alterations of other organs^8,9^. The pathogenic mechanisms responsible for the most severe clinical progression of COVID-19 are yet poorly understood, although they appear to be multifactorial in nature. In this context, a relatively underexplored mechanistic pathway relates to autoimmunity. Autoantibodies that neutralize type-1 interferons have been described in severe adult COVID-19^10^, as have autoantibodies against self-antigens associated with systemic lupus erythematosus and Sjogren’s disease in severe pediatric COVID-19^11^. Additional reports of antiphospholipid autoantibodies have been associated with thrombotic events^12,13^ thereby linking immune dysregulation with thrombosis in severe COVID-19^14^. These observations underscore the urgent need to closely examine the intersection of immunopathology and severe COVID-19, particularly in pulmonary and vascular sites.

In this study, we first sought to detect auto-reactive antibodies in patient plasma using a comprehensive screening approach incorporating diverse and relevant cell types. Plasma samples were obtained from 64 patients hospitalized for COVID-19, including 55 patients with critical illness admitted to the intensive care unit (ICU; COVID ICU) and 9 patients with less severe disease admitted to the regular hospital floor (COVID non-ICU). Plasma was also obtained from 13 critically ill patients without SARS-CoV-2 infection (non-COVID ICU), 9 outpatients with hypergammaglobulinemia (Hyper-γ), and 12 healthy donors (Supplementary Table 1). Samples were screened for the presence of IgA, IgG, and IgM antibodies against 5 human cell types comprising of primary epithelial or endothelial cells of pulmonary, gut, or renal origin, as well as a highly utilized immortalized cell line with a pulmonary endothelial phenotype. Given that these cells have never been exposed to SARS-CoV-2 naïve, antibodies detected in this assay reflect the targeting of self-antigens and are not the consequence of reactivity against SARS-CoV-2 antigens.

Analysis of cells using conventional (Figure 1a) and imaging flow cytometry (Figure 1b–d) revealed the presence of antibodies binding to the plasma membrane. Scored against healthy and non-COVID controls, auto-reactive IgA, auto-reactive IgG and auto-reactive IgM were detected in 28 (51%), 23 (42%), and 51 (93%) out of 55 COVID ICU patients, respectively (Figure 1e). In each reaction, the percentage of cells that stained positively for IgM antibodies was far greater than IgA or IgG, suggesting higher circulating auto-reactive IgM titers. Although COVID ICU patients were associated with higher circulating interleukin-6 (IL-6) and C-reactive protein (CRP) (Supplementary Figure 1a–b), only auto-IgM levels were modestly associated with increased plasma interleukin-6 (IL-6) (ρ=0.29, p=0.0056; Supplementary Figure 2a–b). Of note, most COVID ICU patient plasma showed IgA, IgG, IgM, or a combination of, reactivity with cells of pulmonary origin (Figure 1f). Although a significant percentage of COVID ICU patients had detectable levels of auto-reactive IgA and IgG, we focused on auto-reactive IgM given its substantially higher titers and frequency. Overall, this first set of data revealed that high-titer auto-reactive IgM are frequently detected in patients with severe COVID-19 and that the reactivity is most pronounced against cells of pulmonary epithelial and endothelial origin.

We next sought to understand which auto-antigens are targeted by these circulating auto-reactive IgM in COVID-19 patients. Plasma samples from COVID ICU patients with strong auto-reactive IgM titers (n=5), non-COVID ICU patients (n=3) and healthy controls (n=4) were surveyed in analytical human proteome microarrays (HuProt v4 array). The array epxresses over 21,000 intact proteins, therefore allowing for a thorough and comprehensive investigation of potential binding targets for auto-reactive IgM antibodies. For stringency, a potential binding target was considered for any protein that had a fluorescence signal at least 4 standard deviations (Z-score>4) above the array mean. Additionally, the target had to possess a fluorescence signal at least 2 Z-scores above the same target across all healthy controls. This strict approach resulted in the identification of 260 candidate autoantigens that were uniquely linked to COVID ICU patients (Figure 2a and Supplementary Table 2). Of note, the auto-reactive IgM repertoire in COVID ICU patients is broad, and the candidate targets infrequently overlapped among different patients included in this cohort (Figure 2b). It is very likely, and anticipated, that interrogation of additional plasma samples from patients with severe COVID-19 by proteome microarray would identify further auto-antigen targets, and that the individual antigenic targets are likely less relevant to the disease pathogenesis than the overall abundance, breadth, and tissue specificity of the observed auto-antibodies.

Given the high Z-scores of each candidate target, the auto-reactive IgM antibodies are circulating at robust titers and/or bind with high avidity to the respective targets. We next sought to determine whether the candidate autoantigens were expressed in key tissue types. Using arterial tissues as surrogates for endothelial sites, small intestinal and colonic tissues as surrogates for gastrointestinal sites, as well as renal, neural, and pulmonary sites, we found 226 candidate autoantigens expressed at above-background levels in these cell types (Figure 2c). Importantly, we identified 16 autoantigens associated with the human plasma membrane proteome^15^ and therefore considered these molecules as important prospective candidate targets for circulating pathogenic auto-reactive IgM (Figure 2d). We next investigated whether these proteins shared similar motifs. Although N-linked glycosylation was predicted in 11 candidate autoantigens, heterogeneity in amino acid sequences flanking predicted N-linked glycosylated residues indicated minimal influence of N-linked glycosylation on potential IgM binding motifs (Supplementary Figure 3a). However, an artificial neural network prediction model^16^ revealed extensive O-linked glycosylation for 12 candidate autoantigens (Supplementary Figure 3b–c). Notably, these sites are enriched for proline and serine, which are signs of authentic glycosylation in regions likely to mediate protein-IgM interactions.

The concomitant observations of auto-reactive IgM potentially targeting O-linked glycosylated motifs and high expression of candidate autoantigens in pulmonary sites led us to hypothesize that auto-reactive IgM are a significant contributor to severe COVID-19 disease. To further explore the *in vivo* relationship between auto-reactive IgM and COVID-19 pathophysiology, we first examined post-mortem pulmonary tissue to determine IgM distribution and presence. Immunohistochemical staining of parafin-embedded lung tissue revealed vastly greater IgM binding to alveolar septa and luminal surfaces of three COVID-19 non-survivors, compared to three COVID-19 negative control patients for whom lung tissue was available from cancer-related resection (Figure 3a). It should be noted that some modest IgM deposition in the COVID-19 negative patient controls was expected, as auto-reactive IgMs can develop during lung cancer progression^17^ and/or following radiation therapy^18^. While we cannot formally rule out that the IgM detected in COVID-19^+^ lung tissue are reactive against SARS-CoV-2 surface antigens, the observed staining patterns are not consistent with the distribution patterns observed for SARS-CoV-2 antigens such as the Spike protein^19,20^. Importantly, the extensive IgM staining patterns are at levels at least three times higher than COVID-19 negative controls (Figure 3b), and are not described for other causes of acute respiratory distress^21^. Further histological analysis revealed, in the lung of severe COVID-19 patients, significant alveolar damage and patchy hemorrhage, alongside extensive inflammatory infiltrate breaching the alveolar lumen. Previous studies have linked alveolar damage to dysregulated cytokine release and neutrophil extracellular traps seeded by resident macrophages^22–25^. Yet, these observations could also be linked to auto-reactive IgM, through the capacity of these immunoglobulins to fix complement and induce cytotoxicity. Indeed, staining for complement component 4 (C4d), a marker of complement activation, showed a two-fold increase in COVID-19 patients compared to negative controls (Figure 3c), indicating frequent *in vivo* complement fixation.

Complement-dependent cytotoxicity (CDC) and complement deregulation have been proposed to play a roles in the pathogenesis of ARDS^26^. Additionally, as there is considerable pulmonary microangiopathy observed in severe COVID-19 patients^27,28^, it is conceivable that CDC can precede or even cause the damage to the pulmonary endothelium. Given the observed IgM and C4d binding to pulmonary targets and to confirm that the auto-reactive IgM can mediate CDC, we next tested plasma samples from severe COVID-19 patients for their capability of fixing complement and inducing cytotoxicity *in vitro*. To this end, we investigated patient plasma samples that showed greater than 10% binding to the respective cell type in the screening assay. Interestingly, we consistently observed higher rates of CDC in cells of pulmonary origin (Figure 3d-h). In addition, while non-COVID-19 ICU patient plasma samples induced limited or no cell death, most COVID-19 ICU patients plasma samples induced cell death at frequencies proportional to their measured level of cell binding (Figure 3i). Collectively, these data indicate that auto-reactive IgM present in plasma from severe COVID-19 patients can fix complement and induce cytotoxicity.

The identification of auto-reactive IgM as a potential contributing factor to the pathogenesis of severe COVID-19 has two immediate implications. First, this observation may explain how COVID-19 is disproportionately more serious in the elderly^29^, who typically manifest higher plasma levels of circulating auto-reactive antibodies^30^. This phenomenon would be exacerbated by decreases in functional T follicular helper cells that promote antibody class switching^31^, a process associated with better disease outcomes^32^. Given that IgM levels peak within a week of the clinical onset of COVID-19 and persist at similar levels for weeks thereafter^34^, the elderly face a protracted period where there is steadfast secretion of auto-reactive IgM that maintain relatively low affinity for the same epitope without either switching to alternate antibody class types or undergoing somatic hypermutation and affinity maturation. In this perspective, the elderly may be more prone to severe COVID-19 due to a more protracted exposure to the cytopathic effects of auto-reactive IgM.

Secondly, it is conceivable that this type of immunopathology can be limited by therapeutic interventions that inhibit the IgM-complement axis. In the immediate term, this approach could mitigate the SARS-CoV-2 associated alveolar damage and ARDS^35–37^, and consequently protect against mortality^38^ and/or reduce the need for invasive mechanical ventilation^39^. In the long term, preservation of lung integrity may prevent pathogenic sequelae such as pulmonary fibrosis^40,41^, which diminishes lung function post-recovery^42^. These therapeutic goals could be implemented through the use of immunosuppressants, such as dexamethasone, that can attenuate the production of auto-reactive IgM ^43^, plasma exchange to remove auto-reactive IgM once formed^44^, or to synergize and supplement proposed anti-fibrotic therapies^45^. Alternatively, the complement cascade can be directly inhibited through conestat alfa^46^ or eculizumab^46^, and indeed, both drugs are presently undergoing evaluation through clinical trials to determine eficacy^47^. Optimistically, our findings cast support for interventions that can be readily and swiftly implemented in the clinic to alleviate or prevent serious COVID-19 complications.

In summary, we found that broadly auto-reactive IgM are common in the plasma of patients with severe COVID-19. These auto-reactive antibodies bind pulmonary epithelial and endothelial targets, at which point they can be potent mediators of cytopathicity through the recruitment of complement. Future studies will investigate the relationship between SARS-CoV-2 infection and the emergence of auto-reactive antibodies, and determine whether immunosuppressive therapy can reduce the levels of auto-reactive IgM in plasma and consequently attenuate the clinical severity of COVID-19.

## Methods

### Plasma Samples.

Plasma samples were obtained from discarded clinical specimens at Emory University Hospital or from healthy donors in accordance with protocols approved by Emory’s Institutional Review Board. Patient demographics and characteristics were obtained by electronic chart review as summarized in Supplemental Table 1.

### Cells.

HULEC-5a cells were obtained from the American Type Culture Collection (ATCC) and maintained in MCDB131 Medium (Gibco, Thermo Fisher) supplemented with 10ng/ml epidermal growth factor (Thermo Fisher), 1µg/ml hydrocortisone (Sigma Aldrich), 10mM L-glutamine (Thermo Fisher), and 10% (v/v) FCS (GeminiBio). Primary human small airway epithelial cells (HSAEC) were purchased (Lifeline Cell Technology) and maintained in BronchiaLife Medium (cat. no. LKL-0023, Lifeline Cell Technology). Primary human alveolar epithelial cells (HAEC) and primary human kidney glomerular endothelial cells (HKGEC) were purchased (CellBiologics) and maintained in Complete Human Epithelial Cell Medium (cat. no. H6621, CellBiologics) and Complete Human Endothelial Cell Medium (cat. no. H1168, CellBiologics), respectively. Primary human small intestine microvascular endothelial cells were purchased (Neuromics) and maintained in ENDO-Growth Medium (cat. no. EGK001, Neuromics). All cells were kept at 37°C in a humidified incubator supplemented with 5% CO_2_ and maintained between 50–80% confluence. Primary cells were grown in cell culture flasks coated with gelatin (cat. no. 6950, CellBiologics) and used between 3–7 passages.

### Flow cytometry detection of auto-antibodies.

Plasma aliquots were stored at −80°C and then thawed at 4°C for use in assays. Cells were detached from culture flasks using TrypLE Express reagent (Thermo Fisher) and resuspended in DPBS at a concentration of 5×10^5^ cells/ml. 100µl of each cell suspension was added to 96-well U-bottom plates, and 50µl of patient or healthy donor plasma added and gently mixed. An IgG positive control was performed by adding human anti-CD98 IgG (cat. no. Ab00361–10.0, Absolute Antibody, 2µl) to one well. Plates were transferred to 4°C for one hour, after which cells were washed with cold DPBS and then incubated with an antibody cocktail containing a viability dye (LIVE/DEAD Aqua, Thermo Fisher), anti-CD62E BV605 (cat. no., 563359, BD, 2.5µl), anti-CD54 BV711 (cat. no., 564078, BD, 2.5µl), anti-CD144 BV786 (cat. no., 565672, BD, 2.5µl), anti-CD31 PE (cat. no., 555446, BD, 10µl), anti-human IgG DyLight 650 (cat. no., SA5–10137, Thermo Fisher, 2µl), anti-human IgA FITC (cat. no., A18788, Thermo Fisher, 0.1µl) and anti-human IgM BV650 (cat. no., BioLegend, 314526, 2µl). No-anti-Ig fluorescence minus one controls were also prepared. After one hour at 4°C, cells were washed twice with FACS buffer and then fixed with 1% PFA before analysis on a BD LSRFortessa flow cytometer. For imaging flow cytometry, cells were stained only with anti-IgM BV650 following plasma incubation. Nuclei were stained with NucSpot Live 488 (cat. no. 40081, Biotium, 1:1000). Cells were then fixed in 2% PFA and analyzed on a Luminex Amnis ImageStreamX Mark II flow cytometer.

### IIL-6 ELISA.

Plasma levels of IL-6 were quantified using a Human IL-6 ELISA kit (ab178013, Abcam) and following the manufacturer’s instructions.

### Histology.

Five-micrometer sections from formalin-fixed, parafin-embedded lung tissue sections were tested for IgM expression using a rabbit anti-IgM polyclonal antibody (cat. no. F0203, Agilent, Santa Clara, CA) at 1:400 dilution and for C4d expression using a rabbit anti-C4d polyclonal antibody (cat. no. 04-BI-RC4D, ALPCO Diagnostics, Salem, NH) at 1:100 dilution. IgM staining was performed on a Dako Link48 Autostainer with the EnVision FLEX dual-link system (Dako, Carpinteria, California) after heat-induced epitope retrieval in citrate buffer for 30 minutes. C4d staining was performed on a Leica Bond III automated stainer with the Bond Polymer Refine Detection Kit (Leica Microsystems, Bannockburn, IL) after on-board epitope retrieval using Bond epitope retrieval solution 1 (ER1) for 20 minutes. Images were analyzed in ImageJ using the IHC Image Analysis Toolbox for the enumeration of nuclei, and to identify stained regions. The Color Pixel Counter plugin was further used to quantify the extent of staining in each image.

### Complement Fixing Assay.

Target cells were dissociated from culture flasks by TrypLE Express reagent (Gibco) and resuspended in PBS at a concentration of 1×10^6^ cells/ml. 50µl of the cell suspension was transferred to wells of a 96-well V-bottom plate. 50µl of plasma was added to each well and plates were incubated at 4°C for one hour. 2 non-COVID (ICU) and 2 healthy donor plasma samples without IgM reactivity were selected as controls. Cells were washed with cold DPBS twice and resuspended in 100µl DPBS. 11µl of reconstituted rabbit complement (Low-Tox-M rabbit complement, Cedarlane) was added to each well. To one well, 0.1% Triton X-100 was added to induce cell lysis. Plates were then transferred to a 37°C incubator for two hours. Plates were then centrifuged at 500g for 5 minutes to pellet cells. 50µl of the supernatant was transferred to a flat-bottom 96-well plate in duplicate. 50µl of reconstituted lactose dehydrogenase assay reagent (CyQUANT LDH Cytotoxicity Assay, Invitrogen) was then added to each well, and the plate was subsequently protected from light and left at ambient temperature for 30 minutes, after which 50µl of the included stop solution was added. Absorbance was read at 490nm and 680nm (Varioskan LUX multimode plate reader, Thermo Fisher). Absorbance values at 680nm were subtracted from absorbances at 490nm and duplicate values averaged. Percentage cytotoxicity was calculated by comparing the absorbance values against the lysed-cell and healthy-donor controls.

### Protein Array.

5 COVID-19 (ICU) and 3 non-COVID-19 (ICU) samples characterized as enriched with auto-IgM by the flow cytometry assay described above were submitted alongside 4 randomly chosen healthy control samples to CDI laboratories (Baltimore, MD) for antigen-specificity screening across >21,000 full-length recombinant human protein targets (HuProt v4.0 proteome microarray).

### Gene Expression Analysis.

Tissue-level transcription profiles were based on the Transcript TPMs dataset provided by the GTEx Portal. Subcellular localization data provided by the Human Protein Atlas^15^ guided the identification of plasma membrane proteins. For all analyses, plasma membrane proteins were those defined as ‘Enhanced’ or ‘Supported’ for plasma membrane localization. Visualizations and heatmaps were generated with GraphPad Prism (v9.0) and RStudio Desktop (1.3.959). Predictions of N- and O-linked glycosylated sites were respectively provided by NetNGlyc^48^ and YinOYang servers^16^, and only high-cutoff sites were chosen for further analysis. Amino acid probability graphs were generated with WebLogo 3.

### Statistical Analyses.

GraphPad Prism (v9.0) was used to calculate statistical significances and correlations. Corresponding statistical tests are noted in figure legends.

## Supplementary Material

1

2

3

4

5

## Figures and Tables

**Figure 1:**
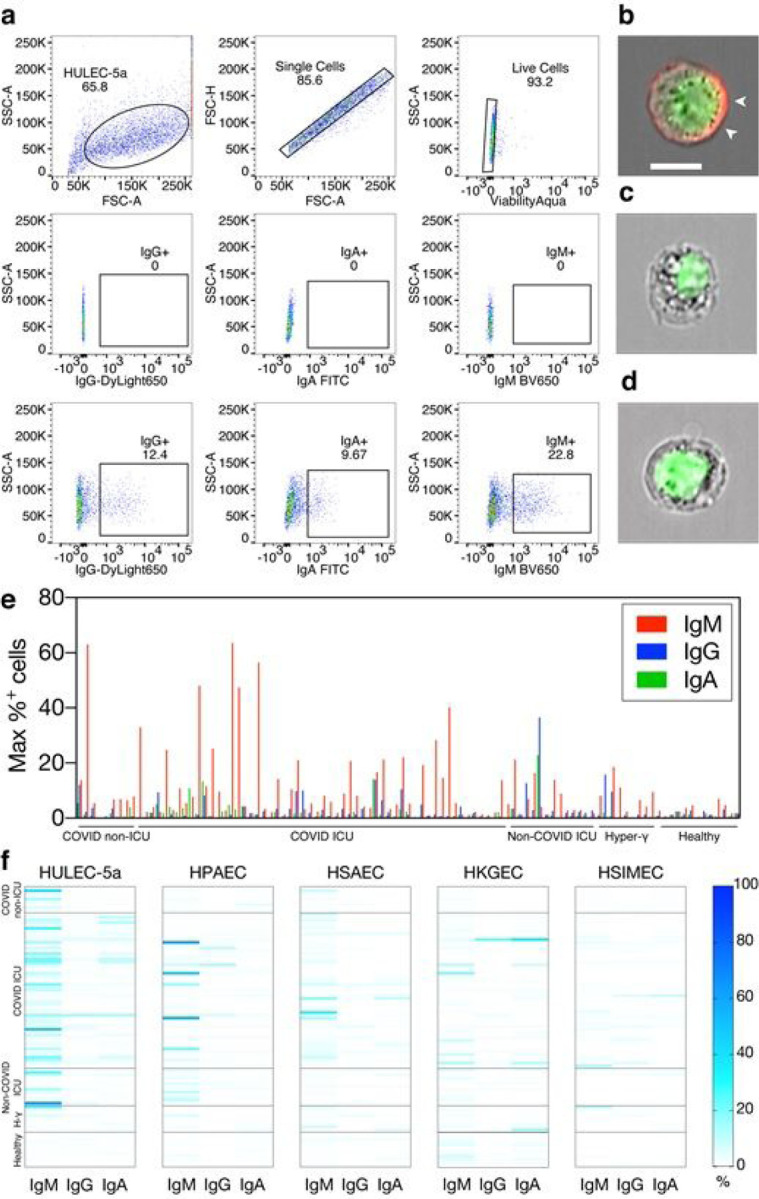
COVID-19 patient plasma contains autoantibodies that bind diverse cell types. a: The presence of auto-Ig was detected in human plasma by flow cytometry. Following initial gating on single and live cells (top row), populations were queried for surface-bound antibodies. Fluorescence minus one (FMO) samples (middle row) and an IgG positive control were used to determine the IgG+ gate (bottom left), whilst gates for IgA+ and IgM+ events were informed by FMO samples and strategic gating to restrict positive events below 2% in at least half and below 10% in all healthy donor samples (bottom middle and bottom right). Representative flow cytometry plots shown. b-d: Imaging flow cytometry detected auto-IgM (pseudocolored red) bound to the plasma membrane of a primary human alveolar epithelial cell (HPAEC) stained with patient plasma containing a high level of auto-IgM (b). This was not observed in cells incubated with patient plasma without HPAEC-reactive auto-IgM (c), or with plasma obtained from a healthy human control (d). Nuclei are pseudocolored green. Distance bar indicates 10µm. IgM-stained plasma membrane indicated by white arrowheads. Representative images shown. e: The maximum observed auto-Ig staining percentage across all cell types, from each patient, are shown. f: Detected auto- Ig levels in specific cell types are shown, per patient. For e and f, The ‘ICU’ label designates non-COVID ICU patients; ‘Hyper-γ’ or ‘H-γ’ label indicates samples from patients with hypergammaglobulinemia. Primary cells used were Human Kidney Glomerular Endothelial Cells (HKGEC), Human Small Airway Epithelial Cells (HSAEC), Human Small Intestinal Microvascular Endothelial Cells (HSIMEC), and Human Pulmonary Airway Epithelial Cells (HPAEC).
