# Supplementary figures and images for: Broad Auto-Reactive IgM Responses Are Common In Critically Ill COVID-19 Patients
Source: Res Sq. 2020 Dec 31:rs.3.rs-128348. Preprint. [Version 1] doi: 10.21203/rs.3.rs-128348/v1 (PMC7781325; doi:10.21203/rs.3.rs-128348/v1)

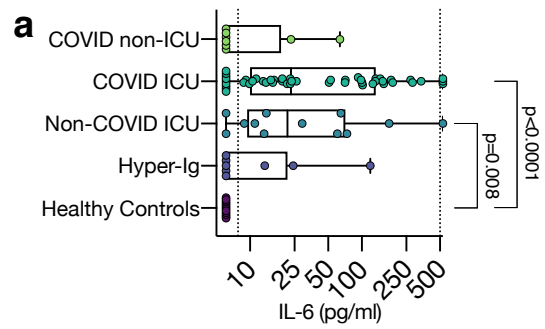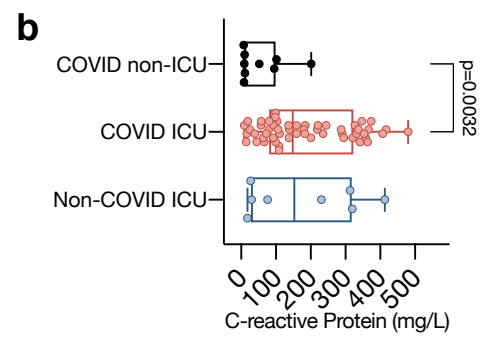

Supplement: 1 [file 69a4ec5d18fc6f1db9b873cf.pdf]

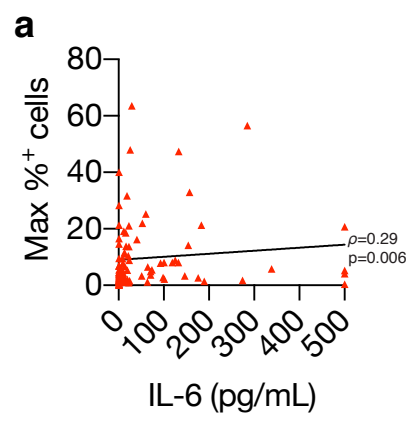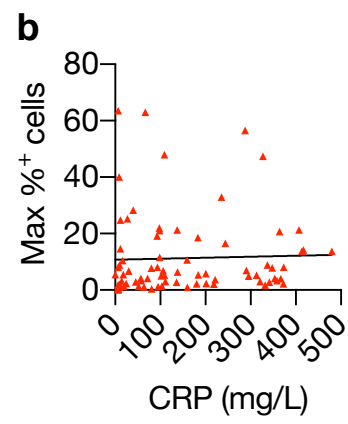

Supplement: 2 [file 4206e68a88f461a2e7efb102.pdf]

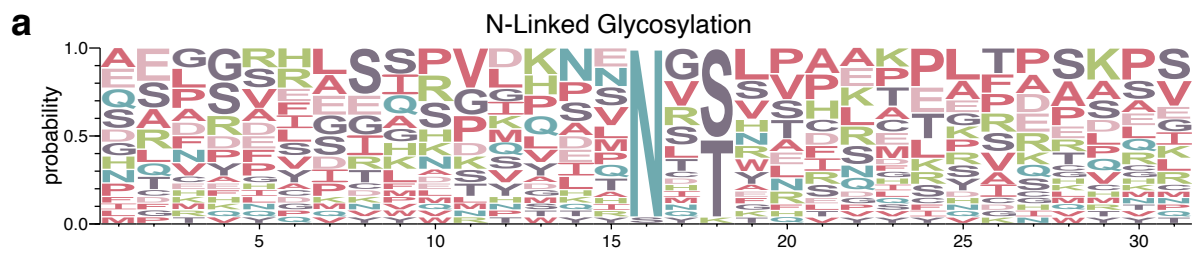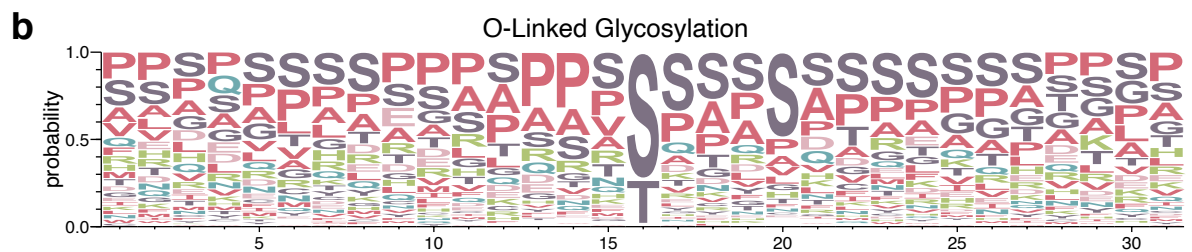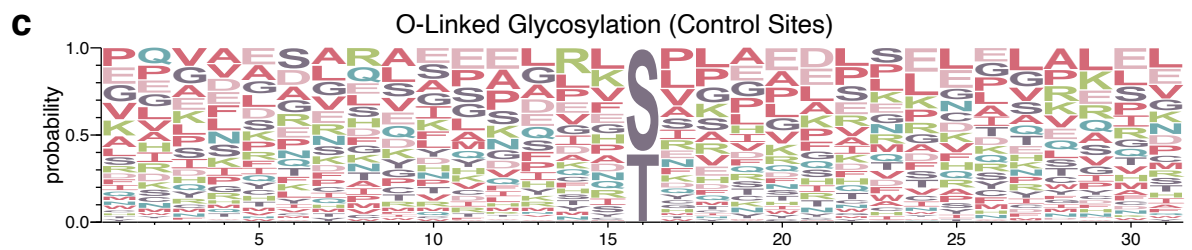

Supplement: 3 [file 46e864fbf8739e0be3595885.pdf]
