# Supplementary material for: Broad Auto-Reactive IgM Responses Are Common In Critically Ill COVID-19 Patients
Source: Res Sq. 2020 Dec 31:rs.3.rs-128348. Preprint. [Version 1] doi: 10.21203/rs.3.rs-128348/v1 (PMC7781325; doi:10.21203/rs.3.rs-128348/v1)
Supplement: 4 [file 6cb5e7c4579ace92cc5c2b21.docx]

Supplementary Table 1: Patient demographics and characteristics.

|  | Median (Range) |
| --- | --- |
| COVID ICU Patients, n=55 |  |
| Age, years | 56 (27-78) |
| Sex, male | 32 (58%) |
| IL-6, pg/ml (normal <5) | 23.3 (<7.8->500) |
| CRP, mg/L (normal <10) | 149 (10-417) |
| Viscosity, cP (normal 1.4-1.8) | 2.2 (1.4-3.6) |
| Non-COVID ICU Patients, n=13 |  |
| Age, years | 58 (24-82) |
| Sex, male | 7 (54%) |
| IL-6, pg/ml (normal <5) | 21.7 (<7.8->500) |
| CRP, mg/L (normal <10) | 272 (18-414) |
| Viscosity, cP (normal 1.4-1.8) | 1.9 (1.6-2) |
| COVID Floor Patients, n=9 |  |
| Age, years | 56 (31-76) |
| Sex, male | 2 (22%) |
| IL-6, pg/ml (normal <5) | <7.8 (<7.8-64.0) |
| CRP, mg/L (normal <10) | 11.4 (6.9-202) |
| Non-COVID Hypergammaglobulinemia Outpatients, n=9 |  |
| Age, years | 66 (52-92) |
| Sex, male | 5 (55%) |
| IL-6 | <7.8 (<7.8-118.8) |
| Viscosity, cP (normal 1.4-1.8) | 2.3 (1.9-5.1) |
| Healthy Controls, n=12 |  |
| Age, years | 41 (27-51) |
| Sex, male | 5 (42%) |
| IL-6, pg/ml (normal <5) | <7.8 (<7.8) |
